# HBV reactivation in HCC patients following TACE: risk factors, antiviral therapy impact, and survival analysis

**DOI:** 10.3389/fonc.2026.1818955

**Published:** 2026-04-28

**Authors:** Hao Zhang, Jia-Hang Du, Zhi-Yong Lu, De-Di Wu, Lin Ma, Jian-Yong Yang, Yong-Hui Huang, Ying−Qiang Zhang

**Affiliations:** 1 Department of Interventional Radiology, The Seventh Affiliated Hospital of Sun Yat-sen University, Shenzhen, Guangdong, China; 2 Department of Interventional Radiology, The First Affiliated Hospital, Sun Yat-Sen University, Guangzhou, China

**Keywords:** antiviral therapy, hepatitis B virus, hepatocellular carcinoma, reactivation, risk factors, survival analysis, transcatheter arterial chemoembolization

## Abstract

**Background:**

Hepatitis B virus (HBV) reactivation after transcatheter Arterial Chemoembolization (TACE) can lead to severe complications and affect prognosis. Current research on HBV reactivation following TACE is limited and inconsistent. Long-term prognostic data on antiviral therapy (AVT) in patients undergoing TACE are also scarce.

**Purpose:**

This study aimed to evaluate the risk factors for HBV reactivation in hepatitis B surface antigen (HBsAg) positive Hepatocellular carcinoma (HCC) patients following TACE and the impact of prophylactic AVT on HBV reactivation and survival outcomes.

**Patients and methods:**

This retrospective study included HBsAg-positive, treatment-naive HCC patients who underwent TACE between December 2020 and December 2024. Patient baseline characteristics, TACE procedures, and follow-up data were collected. HBV reactivation was defined as a ≥2 log IU/mL increase in HBV DNA levels from baseline or HBsAg seroconversion. Prophylactic AVT was defined as anti-HBV drug administration before or during TACE, continued throughout follow-up. Survival outcomes were assessed using the Kaplan-Meier method, log-rank test and Cox proportional hazards regression analyses.

**Results:**

A total of 168 patients were enrolled. HBV reactivation occurred in 32 patients (19.0%). Multivariate analysis identified the absence of prophylactic AVT (odds ratio [OR] 3.56, 95% CI 1.55-8.18, P = 0.003) and chemotherapeutic agents (>2 agents) (OR 2.79, 95% CI 1.09-7.14, P = 0.032) as independent risk factors for HBV reactivation. Unadjusted Kaplan–Meier analysis showed that patients without HBV reactivation had significantly better OS (P = 0.036). Multivariable Cox proportional hazards regression analysis confirmed that HBV reactivation (hazard ratio [HR]=1.43, 95%CI:0.90–2.28, P = 0.047) and macrovascular invasion (HR = 1.69, 95%CI:1.14–2.51, P = 0.017) were independent predictors of poor OS, whereas prophylactic AVT was not significantly associated with OS (HR = 0.75, 95%CI:0.50–1.14, P = 0.182).

**Conclusion:**

In HBsAg-positive HCC patients undergoing TACE, the absence of prophylactic AVT and chemotherapeutic agents (>2 agents) are significant risk factors for HBV reactivation. HBV reactivation and macrovascular invasion are independent predictors of poor OS, while prophylactic AVT shows no significant association with OS. These findings provide real-world evidence for optimizing the clinical management of HBV-related HCC patients undergoing TACE.

## Introduction

Hepatocellular carcinoma (HCC) ranks as the sixth most prevalent cancer globally and the third leading cause of cancer-related deaths (1–3). Hepatitis B virus (HBV) infection stands as a primary etiological factor for liver cancer, significantly elevating the mortality risk in patients with cirrhosis and HCC (4, 5). For patients ineligible for surgical resection or liver transplantation, interventional therapy is pivotal, with transcatheter arterial chemoembolization (TACE) being the most widely used intervention for Barcelona Clinic Liver Cancer (BCLC) stage B patients (6–8).

A well-documented concern is HBV reactivation in patients receiving cytotoxic chemotherapy or immunosuppressants for hematological malignancies and solid tumors (9, 10). This reactivation can trigger asymptomatic elevations in HBV DNA and alanine aminotransferase (ALT) levels, as well as severe outcomes such as hepatitis, liver failure, and even death—ultimately disrupting tumor treatment and worsening patient prognosis (11, 12). Given the high prevalence of HBV infection in HCC patients, TACE-induced HBV reactivation is theoretically a critical clinical issue. However, relevant research remains limited and inconsistent.

Key challenges in the field include the lack of standardized diagnostic criteria for HBV reactivation, leading to variable definitions across studies (13–15). Some studies adopt a less rigorous definition (e.g., a ten-fold increase in HBV DNA), which may overstate the impact of irregular viral fluctuations and yield false-positive results (16–18). Additionally, many studies focus solely on data from a single TACE session, overlooking the continuous nature of HCC treatment (17, 18). While numerous studies have demonstrated that antiviral therapy (AVT) improves postoperative outcomes, including disease-free survival (DFS) and overall survival (OS) in HBV-related HCC patients after curative resection (19–21), evidence regarding the long-term prognostic value of AVT in TACE-treated patients is scarce. To our knowledge, only Jang et al. reported in a large cohort study that prophylactic AVT was significantly associated with improved long-term survival in this population (22), but this finding requires further validation.

Against this backdrop, we conducted this retrospective study to address these existing gaps. The primary objectives were to evaluate the risk factors for HBV reactivation in HBsAg-positive HCC patients undergoing TACE (reflecting real-world treatment protocols, including multiple TACE sessions) and to assess the impact of prophylactic AVT on HBV reactivation and survival outcomes. This study aims to provide robust real-world evidence to optimize the clinical management of HBV-related HCC patients undergoing TACE.

## Patients and methods

### Patients

The protocol was approved by the Ethics Committees of the Hospital. Written informed consent was obtained from every patient, and the study was conducted in accordance with the Declaration of Helsinki. This research included treatment-native patients with HBsAg-positive HCC who underwent TACE between December 2020 and December 2024. HCC diagnosis was established based on the criteria outlined in the clinical practice guidelines of the European Association for the Study of the Liver (EASL) (23). The inclusion criteria were as follows: (a) patients with histologically confirmed HCC or diagnosed with HCC based on two imaging modalities; (b) liver function classified as Child-Pugh class A or B; (c) Eastern Cooperative Oncology Group performance status (ECOG-PS) of 0 or 1; (d) HBsAg positivity; and (e) expected survival of more than three months. The exclusion criteria were: (a) missing key data, including baseline HBV DNA levels, antiviral treatment information, HBV DNA level/liver function during follow-up and insufficient survival follow-up; (b) positive markers for other hepatotropic viruses or HIV infection; (c) presence of other malignancies or severe comorbidities, including organ dysfunction and hepatic or renal failure; and (d) history of treatment for other malignancies.

### Chemoembolization procedure

All patients underwent either conventional lipiodol TACE (c-TACE) or drug-eluting bead TACE (d-TACE). The procedures were performed by senior radiologists with over a decade of experience in interventional radiology. The procedure involved inserting a 5-F catheter into the celiac and superior mesenteric arteries, followed by angiography to evaluate the tumor vascular supply and portal vein patency. Each procedure included super-selective catheterization of the tumor-feeding arteries using a microcatheter. Chemotherapeutic agents such as Epirubicin (20–40 mg), oxaliplatin (100–200 mg), irinotecan hydrochloride (80–240 mg), mitomycin (4 mg), and 5-fluorouracil (500 mg) were selected based on tumor characteristics, patient condition, and clinical factors. The selection and dosage were individualized for each patient in the study. For c-TACE, a 2–10 mL emulsion of Lipiodol mixed with chemotherapeutic agents was infused into the hepatic tumor via a microcatheter. Gelatinous sponge particles were used for embolization until blood flow stasis was achieved in the tumor-feeding arteries. The d-TACE protocol involved the super-selective injection of drug-eluting beads loaded with Epirubicin (40–60 mg). If the tumor-feeding arteries were not completely embolized, additional embolization with gelatinous sponge particles was performed. The TACE regimen followed an on-demand strategy (18). Repeat TACE treatments were administered when viable tumors or new intrahepatic lesions were visible on enhanced CT or MR imaging and the patient’s liver function was preserved (Child-Pugh Class A or B).

### Definitions

Hepatitis B reactivation is defined as (a) an increase in HBV DNA level by ≥2 log (100-fold) IU/mL compared to baseline or seroconversion from negative to positive for Hepatitis B surface antigen (HBsAg) or (b) for patients with previously undetectable HBV DNA levels, a level of HBV DNA ≥3 log (1000) IU/mL (24). OS was defined as the time from the first TACE to death from any cause, or the last follow-up if the patient was still alive.

### Follow-up

Participants underwent follow-up evaluations every 3–6 weeks for laboratory tests, including complete blood count, liver function, coagulation profile, tumor markers (AFP), serological markers for Hepatitis B, and HBV DNA (monitored every 1–3 months). Tumor characteristics were evaluated using CT, MRI, or ultrasonography (USG) to assess changes in tumor features, enhancement during the arterial phase, and lipoid deposition. TACE was repeated until tumor stability or progression, intolerable toxicity, or patient refusal occurred. The endpoint of the observation was OS.

### Statistical analysis

Statistical analyses were conducted using SPSS version 25.0 software. Categorical variables were expressed as frequencies or percentages, and continuous numerical variables were described using median or mean values with standard deviations (x ± s). Pearson’s chi-squared test was used to analyze the characteristics of categorical variables across groups, and independent samples t-tests were used to compare the characteristics of continuous numerical variables among groups. Logistic binary regression analysis was used to investigate the factors associated with HBV reactivation. The multivariate model included only factors with p-values less than 0.05 in the univariate model. Regression analysis results were presented as odds ratios (OR) with 95% confidence interval (CI) and p-values. Survival probabilities were estimated using the Kaplan-Meier method, and survival curves were compared between groups by log-rank test. Statistical significance was set at P < 0.05. Univariate and multivariable Cox proportional hazards regression analyses were performed to identify prognostic factors for overall survival. Variables with P < 0.10 in the univariate Cox model were entered into the multivariable model.

## Results

### Study population

**Figure 1:**
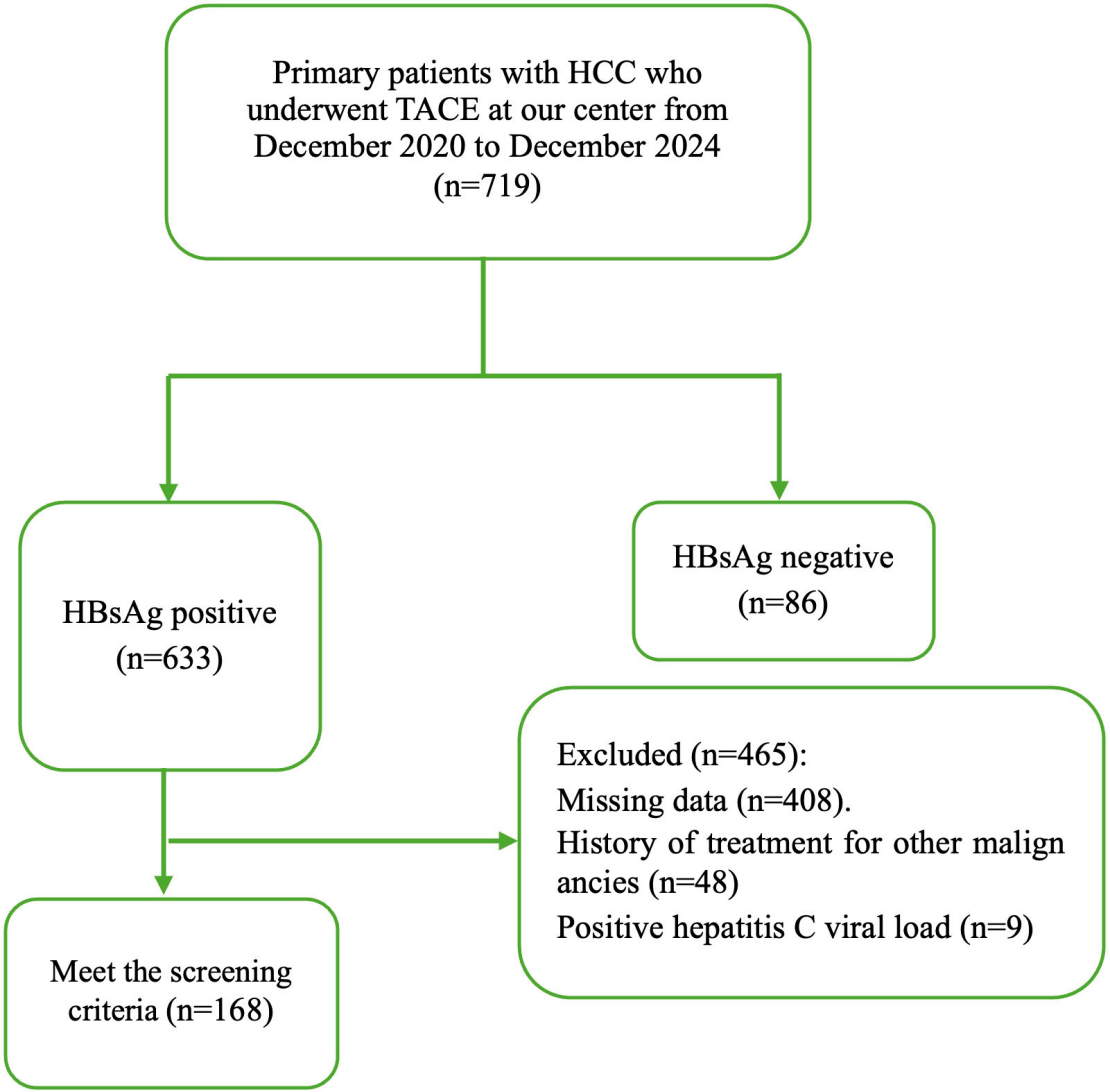
Flowchart depicting patient deposition. HCC, hepatocellular carcinoma; TACE, transcatheter arterial chemoembolization; HBsAg, hepatitis B surface antigen.

A total of 168 HBsAg-positive patients with HCC were enrolled in the study. **Figure 1** illustrates the patient-selection process. The average age of all patients was 50.71 ± 11.75 years old. The average number of TACE procedures performed on all patients was 3.1. A total of 146 (86.9%) patients underwent c-TACE, whereas 22 (13.1%) received d-TACE. **Table 1** presents a detailed overview of the general clinical and TACE treatment characteristics.

**Table 1:** Baseline clinical and treatment characteristics of patients.

Characteristics	Description
Sex (males: females)	159:9
Age (years)	50.71 ± 11.75
WBC (10^9^/L)	6.29 ± 2.37
PLT (10^9^/L)	179.67 ± 86.47
ALB (g/L)	36.70 ± 5.42
TBIL (umol/L)	20.43 ± 13.54
ALT (U/L)	54.14 ± 49.98
AST (U/L)	74.50 ± 57.55
PT (s)	12.86 ± 1.17
Tumor number (≤3: >3)	74:94
Tumor diameter (≤5cm: >5cm)	46:122
Macrovascular invasion (no: yes)	127:41
Extrahepatic spread (no: yes)	149:19
BCLC stage (A: B:C)	26:92:50
AFP (<400 ng/mL: ≥400 ng/mL)	83:85
HBV DNA (<10^4^ IU/mL: ≥10^4^ IU/mL)	83:85
HBeAg (negative: positive)	122:46
Anti-HBe (negative: positive)	121:47
Child-Pugh grade (A:B)	130:38
Prophylactic AVT^a^ (no: yes)	53:115
Type of TACE (c-TACE: d-TACE)	146:22
Cycles of TACE (≤3: >3)	77:91
Resection after TACE (no: yes)	150:18
Subsequent systemic therapy^b^ (no: yes)	16:152
Microwave ablation after TACE (no: yes)	99:69
Chemotherapeutic agents (epirubicin only:>2 agents^c^)	65:103

Data are numbers of patients; ^a^Prophylactic AVT is defined as the administration of anti-HBV drugs prior to or concurrent with TACE intervention, with patients continuing antiviral medication throughout follow-up; ^b^Subsequent systemic therapy, including targeted agents and immunotherapy; ^c^Chemotherapeutic agents (>2 agents) defined as the use of two or more different chemotherapeutic drugs in a single TACE session; ALB, albumin; ALT, alanine transaminase; AST, aspartate transaminase; AFP, alpha-fetoprotein; AVT, antiviral therapy; c-TACE, conventional lipiodol transcatheter arterial chemoembolization; d-TACE, drug-eluting bead transcatheter arterial chemoembolization; HBeAg, hepatitis B e antigen; HBV DNA, hepatitis B virus deoxyribonucleic acid; PLT, platelet; PT, prothrombin time; TBIL, total bilirubin; WBC, white blood cell; BCLC, Barcelona Clin Liver Cancer.

### Risk factor analysis for HBV reactivation

HBV reactivation occurred in 32 (19.0%) of the 168 patients during follow-up. **Table 2** shows that baseline HBV DNA levels less than 10^4 IU/mL, chemotherapeutic agents (>2 agents) and not receiving prophylactic AVT were significantly associated with HBV reactivation (P < 0.05). However, multivariate regression analysis identified only the absence of prophylactic antiviral therapy (OR, 3.56; 95% CI, 1.55-8.18, P = 0.003) and chemotherapeutic agents (>2 agents) (OR, 2.79; 95% CIs:1.09-7.14, P = 0.032) as significant risk factors for HBV reactivation.

**Table 2:** Univariate and multivariate logistic regression analysis of risk factors for hepatitis B virus (HBV) reactivation.

Variable	Univariate Analysis		Multivariate Analysis	
	OR (95% CI)	P	OR (95% CI)	P
Age (>51)	0.78 (0.36~1.69)	0.525		
WBC (10^9^/L)	1.02 (0.87~1.20)	0.804		
PLT (10^9^/L)	1.00 (0.99~1.00)	0.935		
ALB (g/L)	1.04 (0.96~1.12)	0.361		
TBIL (umol/L)	0.98 (0.94~1.02)	0.291		
ALT (U/L)	1.0 (0.98~1.01)	0.560		
AST (U/L)	0.99 (0.99~1.00)	0.200		
PT (s)	0.77 (0.53~1.11)	0.156		
APTT (s)	0.96 (0.87~1.06)	0.428		
Tumor number (>3)	0.64 (0.29~1.38)	0.252		
Tumor diameter (>5cm)	0.56 (0.25~1.25)	0.157		
Macrovascular invasion (yes)	1.13 (0.52~2.43)	0.764		
Extrahepatic spread (yes)	0.47 (0.10~2.13)	0.325		
BCLC stage (B)	0.69 (0.24~2.01)	0.496		
AFP (>400ng/ml)	0.97 (0.45~2.10)	0.940		
Child-Pugh grade (B)	0.75 (0.28~1.98)	0.562		
HBeAg (Positive)	0.43 (0.15~1.19)	0.105		
Anti-HBe (Positive)	0.41 (0.15~1.15)	0.091		
HBV-DNA (≥10^4^IU/mL)	0.44 (0.20~0.98)	**0.045**	0.56 (0.24~1.29)	0.173
Prophylactic AVT (No)	3.71 (1.67~8.23)	**0.001**	3.56 (1.55~8.18)	**0.003**
Type of TACE (d-TACE)	1.73 (0.62~4.85)	0.296		
Cycles of TACE (≥3)	1.53 (0.69~3.37)	0.295		
Resection after TACE (yes)	2.39 (0.82~6.93)	0.111		
Subsequent systemic therapy (yes)	1.72 (0.37~7.98)	0.488		
Microwave ablation after TACE	2.14 (0.98~4.67)	0.055		
Chemotherapeutic agents (>2 agents)	2.66 (1.08~6.56)	**0.034**	2.79 (1.09~7.14)	**0.032**

Bold values indicate statistical significance (P < 0.05).

Variables with P<0.05 in univariate analysis were included in multivariate analysis. Bold values indicate statistical significance (P<0.05). In the multivariate logistic regression model, 32 HBV reactivation events and 3 variables were included, with 10.7 events per variable, indicating a low risk of model overfitting.

### Survival analysis

**Figure 2:**
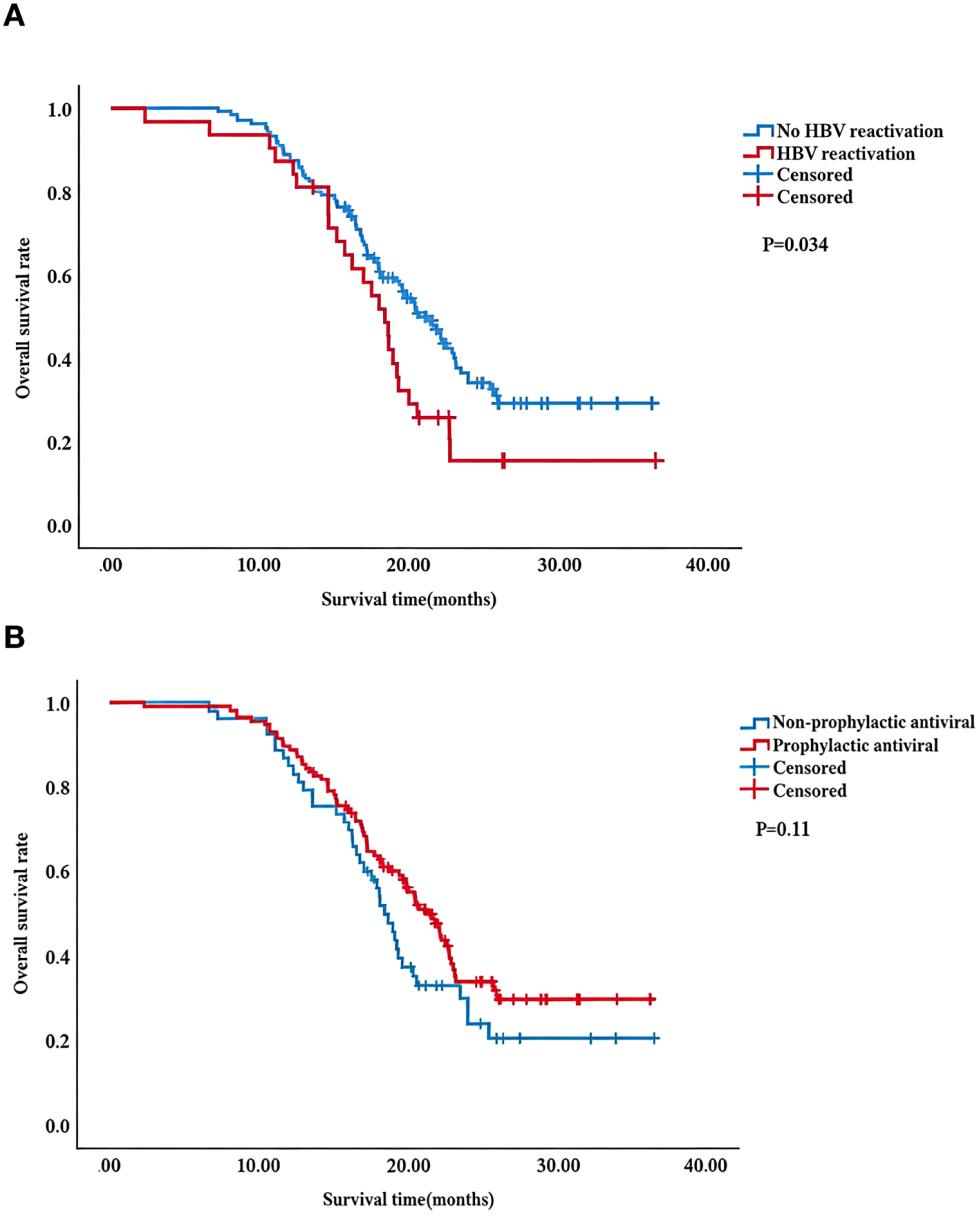
Overall survival analysis. hepatitis B virus (HBV) reactivation and no HBV reactivation groups **(A)**, Overall survival (OS) comparison between prophylactic antiviral therapy (AVT) and non- prophylactic antiviral groups **(B)** using Kaplan-Meier method. OS was defined as the time from the first TACE to death from any cause, or the last follow-up if the patient was still alive. The difference was significant if P<0.05 by the log-rank test.

Unadjusted Kaplan–Meier curves were plotted for the primary study variables. As depicted in **Figure 2A**, patients without HBV reactivation exhibited superior OS compared to those with HBV reactivation (P = 0.036). The median OS for patients without HBV reactivation was 19.60 months (95% CI, 8.86–33.20 months), whereas that for patients with HBV reactivation was 18.20 months (95% CI, 5.67–28.60 months). However, there was no significant difference in OS between the prophylactic antiviral (median, 19.80 months; 95% CI, 8.52–31.80 months) and non-prophylactic antiviral groups (median, 18.0 months; 95% CI, 8.20–33.30 months) (p=0.11) (**Figure 2B**). Multivariable Cox proportional hazards regression analysis confirmed that HBV reactivation (HR = 1.43, 95%CI:0.90–2.28, P = 0.047) and macrovascular invasion (HR = 1.69, 95%CI:1.14–2.51, P = 0.017) were independent predictors of poor OS, whereas prophylactic AVT was not significantly associated with OS (HR = 0.75, 95%CI:0.50–1.14, P = 0.182).

## Discussion

HCC is closely linked to HBV infection, and TACE remains a cornerstone of interventional therapy for unresectable HCC. However, TACE-related HBV reactivation poses a significant threat to treatment outcomes and patient prognosis, making it a critical clinical concern that requires in-depth exploration.

Cytotoxic chemotherapy drugs used in TACE, such as epirubicin, are known to potentially induce HBV reactivation. Existing studies suggest two primary mechanisms underlying this phenomenon: first, extensive hepatocyte damage caused by TACE and subsequent liver regeneration processes may disrupt the stable state of HBV, promoting viral replication (25–27). second, chemotherapeutic agents can induce immune suppression by exhausting B and T cells, which impairs the body’s ability to control HBV and disrupts immune tolerance within tumors (28, 29).This suppression further downregulates the expression of antiviral cytokines like IFN-γ and TNF-α, creating a favorable microenvironment for HBV reactivation. Our multivariate regression analysis identified chemotherapeutic agents (>2 agents) as an independent risk factor for HBV reactivation (OR = 2.79, 95% CI = 1.09-7.14, P = 0.032), which differs from the findings of Lao et al (17). who reported no significant association between combination chemotherapy and HBV reactivation. This discrepancy may be attributed to the fact that Lao et al.’s study only included patients who underwent a single TACE session, resulting in lower cumulative chemotherapy intensity and milder immune suppression compared to our cohort, where patients received an average of 3.1 TACE procedures. The cumulative immunosuppressive effect of multiple TACE sessions, especially with chemotherapeutic agents (>2 agents) that exert stronger immunosuppressive effects than single-agent epirubicin, likely contributes to the higher reactivation risk observed in our study. In addition, the wide 95% confidence interval for chemotherapeutic agents (>2 agents) (1.09–7.14) in the multivariate model warrants cautious interpretation of this result. Regarding TACE modalities, our study included 146 cases of conventional lipiodol TACE (c-TACE) and 22 cases of drug-eluting bead TACE (d-TACE). Theoretically, d-TACE offers sustained local release of chemotherapeutic agents, which may reduce systemic immunosuppression and thus the risk of HBV reactivation (30). However, due to the small sample size of d-TACE patients (13.1%), we did not observe a statistically significant difference in reactivation rates between the two groups, highlighting the need for larger-scale studies to validate this hypothesis.

Chronic hepatitis B (CHB) AVT has established clear clinical guideline consensus. The latest Chinese Guidelines for the Prevention and Treatment of Chronic Hepatitis B explicitly recommend AVT for most CHB patients to inhibit HBV replication, alleviate liver inflammation, and prevent disease progression to cirrhosis, HCC, or liver failure (31). Notably, HBV-related HCC is listed as a key indication for AVT, with initiation recommended regardless of baseline HBV DNA levels or transaminase status (31), which aligns with the core spirit of international guidelines such as the AASLD 2018 guidance (24) while being more tailored to China’s context of high HBV incidence and a large HCC patient population. Our study provides real-world evidence supporting the implementation of these guidelines in the TACE setting: TACE-induced ischemia-reperfusion injury and chemotherapy-mediated immunosuppression disrupt the immune balance of latent HBV, creating an “environment prone to reactivation” (28, 29), and prophylactic AVT effectively mitigates this risk. Specifically, the reactivation rate was significantly lower in patients who received prophylactic AVT (12.20% [14/115]) compared to those who did not (34% [18/53], P = 0.001), and multivariate analysis confirmed non-receipt of prophylactic AVT as a strong independent risk factor (OR = 3.56, 95% CI = 1.55-8.18, P = 0.003). These findings are consistent with most previous studies: a meta-analysis of TACE studies showed that 216 of 226 patients with HBV reactivation had not received prophylactic AVT (32), and similar protective effects of AVT have been reported in HAIC and RFA studies (33, 34). In contrast, Shao et al. (35) found no significant difference in reactivation rates between AVT and non-AVT groups, which may be attributed to antiviral drug resistance among their patients.

Notably, despite the proven efficacy of AVT and clear guideline recommendations, our cohort still had a 19.0% HBV reactivation rate—pointing to a significant gap between clinical practice and guidelines. This gap is driven by three key factors: first, underutilization of prophylactic AVT (only 68.5% of eligible patients received it), stemming from clinical cognitive biases (overreliance on baseline HBV DNA levels, neglect of TACE-induced immunosuppression, and insufficient attention to low viral load [<10^4^ IU/mL] patients), inadequate institutional processes (lack of standardized pre-TACE HBsAg/HBV DNA screening-hepatology consultation-AVT initiation pathways), and patient-level barriers (tumor treatment prioritization, poor long-term adherence, economic concerns); second, a reactive treatment bias—most non-prophylactic AVT patients initiated therapy only after HBV DNA elevation, which fails to prevent initial hepatocellular damage or TACE disruptions, reflecting insufficient interdisciplinary collaboration; third, non-standard treatment duration, with some patients discontinuing AVT independently post-TACE despite guidelines recommending lifelong therapy for HBsAg-positive HCC patients (31, 36). Addressing these gaps requires process-oriented and interdisciplinary interventions: establishing standardized TACE-HBV management pathways (mandatory screening, hepatology consultation, AVT initiation, long-term follow-up), strengthening inter-departmental collaboration, and improving patient adherence through education to ensure guideline recommendations translate into clinical practice.

Traditionally, high baseline HBV DNA levels have been considered a key risk factor for HBV reactivation (37, 38). However, in our study, while univariate analysis showed a correlation between HBV DNA levels and reactivation (P = 0.045), multivariate logistic regression did not identify HBV DNA as an independent predictor (P>0.05). This discrepancy may be explained by differences in study design and patient populations. Previous studies primarily included patients who did not receive prophylactic AVT, whereas in our cohort, 76.5% of patients with HBV DNA ≥10^4^ IU/mL received prophylactic AVT, compared to only 60.2% of those with HBV DNA <10^4^ IU/mL (P = 0.024). The lower AVT utilization rate in the low viral load group may have contributed to their higher reactivation risk observed in univariate analysis, indicating that TACE-related stress overrides the impact of baseline viral load. Thus, our findings suggest that prophylactic AVT should be administered to all HBsAg-positive HCC patients undergoing TACE, regardless of baseline HBV DNA levels.

HBV reactivation has been reported to negatively impact treatment outcomes and prognosis (39), leading to asymptomatic increases in HBV DNA and ALT levels, severe hepatitis, liver failure, and even death (11, 12), which can result in delays or premature interruptions in cancer treatment. Consistent with these reports, our study found that patients without HBV reactivation had significantly better OS than those with reactivation (median OS: 19.60 months vs. 18.20 months, P = 0.036), and multivariable Cox proportional hazards regression analysis confirmed that HBV reactivation (HR = 1.43, 95% CI: 1.03–2.28, *P* = 0.047) was one of independent predictors of poor OS. By contrast, prophylactic AVT was not independently associated with OS [P = 0.11 in unadjusted log-rank test; P = 0.183 (HR = 0.75, 95% CI: 0.50–1.14) in adjusted multivariable Cox model]. It was contrary to the findings of Jang et al. (22) who reported that prophylactic AVT significantly prolonged survival in a large cohort of 1084 HBV-related HCC patients undergoing TACE. Theoretically, NAS therapy can promote viral clearance, suppress hepatic inflammation, alleviate chronic liver injury, and reverse fibrosis (36, 40), which may preserve liver function and enable patients to tolerate additional cycles of anticancer therapy, thereby extending survival. We cautiously propose that the lack of a significant association between prophylactic AVT and OS may be due to the following reasons: First, in our multivariable Cox model, macrovascular invasion was identified as a strong and independent prognostic factor for OS (HR = 1.69, 95%CI:1.14–2.51, P = 0.017). This powerful tumor-related determinant exerts an overwhelming impact on patient survival and may mask or attenuate the potential survival benefit of prophylactic antiviral intervention. Second, Jang et al.’s control group included patients who never received AVT, which is inconsistent with current clinical practice where AVT is widely used. in our study, the non-prophylactic AVT group primarily received delayed AVT (initiated after HBV DNA elevation), which may have mitigated the adverse effects of reactivation and reduced the survival difference between the two groups. Third, the relatively small sample size and retrospective design may have limited statistical power to detect a potential survival advantage of prophylactic AVT. Therefore, while prophylactic AVT effectively prevents HBV reactivation, its impact on long-term OS warrants further validation in large-scale prospective studies. Practice.

This study has several limitations. First, it is a retrospective study, which inherently introduces potential selection bias. Decisions regarding the administration of prophylactic AVT and choice of chemotherapy regimen were based on clinician judgment and patient characteristics rather than randomization, which may have influenced the association between AVT and survival outcomes. Second, a substantial number of patients were excluded due to incomplete antiviral treatment records, missing baseline HBV DNA data, and loss to follow-up within 1–3 months post-TACE, reducing the effective sample size and potentially introducing selection bias. Sensitivity analyses for missing data were not performed due to data limitations, which may affect the robustness of our risk factor analyses. Third, the sample size of d-TACE patients was small, precluding meaningful subgroup analysis to compare reactivation risks between TACE modalities. Fourth, we did not collect HBV genotype data, which has been shown to influence reactivation risk (41), limiting our ability to conduct genotype-specific risk stratification in the context of precision medicine.

## Conclusion

In summary, our study confirms chemotherapeutic agents (>2 agents) and non-receipt of prophylactic AVT are independent risk factors for HBV reactivation in HBsAg positive HCC patients undergoing TACE, and prophylactic AVT effectively prevents or delays HBV reactivation. HBV reactivation and macrovascular invasion were independent predictors of poor OS, while prophylactic AVT showed no significant association with OS. The gap between clinical practice and guidelines underscores the need for standardized TACE-HBV management. Future large-scale prospective studies are warranted to validate our findings and optimize patient care.

Practice labor center.

## Data Availability

The original contributions presented in the study are included in the article/Supplementary Material. Further inquiries can be directed to the corresponding author.
